# Molnupiravir maintains antiviral activity against SARS-CoV-2 variants and exhibits a high barrier to the development of resistance

**DOI:** 10.1128/aac.00953-23

**Published:** 2023-12-04

**Authors:** Julie M. Strizki, John M. Gaspar, John A. Howe, Beth Hutchins, Hiroshi Mohri, Manoj S. Nair, Keith C. Kinek, Philip McKenna, Shih Lin Goh, Nicholas Murgolo

**Affiliations:** 1 Merck Research Laboratories (MRL), Merck & Co., Inc., Rahway, New Jersey, USA; 2 Aaron Diamond AIDS Research Center, Columbia University Medical Center, New York, New York, USA; IrsiCaixa Institut de Recerca de la Sida, Barcelona, Barcelona, Spain

**Keywords:** COVID-19, molnupiravir, N-hydroxycytidine, antiviral resistance

## Abstract

Molnupiravir, an oral prodrug of N-hydroxycytidine (NHC), previously demonstrated broad *in vitro* antiviral activity against multiple RNA viruses and has shown a high barrier to the development of resistance. Here, we present the antiviral activity of NHC against recent SARS-CoV-2 variants and the results of resistance selection studies to better understand the potential for viral resistance to NHC. NHC activity against SARS-CoV-2 variants omicron (BA.1, BA.1.1, BA.2, BA.4, BA.4.6, BA.5, BQ.1.1, XBB.1, and XBB.1.5), alpha (B.1.1.7), beta (B.1.351), gamma (P.1), delta (B.1.617.2), lambda (C.37), and mu (B.1.621) was evaluated in Vero E6 cells using cytopathic effect assays. Resistance selection studies were performed by passaging SARS-CoV-2 (WA1) in the presence of NHC or a 3C-like protease inhibitor (MRK-A) in Vero E6 cells. Supernatants from cultures exhibiting a cytopathic effect score of ≥2 were re-passaged, and IC_50_ values were estimated. Whole-genome deep sequencing was performed on viral RNA isolated at each passage. NHC demonstrated similar potency against all SARS-CoV-2 variants evaluated. No evidence of SARS-CoV-2 phenotypic or genotypic resistance to NHC was observed following 30 passages. A random pattern of nucleotide changes was observed in NHC cultures, consistent with the drug’s mechanism of action. In contrast, resistance was readily selected in all three MRK-A control cultures with the selection of a T21I substitution in the 3C-like protease. In conclusion, molnupiravir maintains antiviral activity across all major SARS-CoV-2 variants. Furthermore, no evidence of viral resistance to NHC was observed, supporting previous reports that NHC has a high barrier to developing resistance.

## INTRODUCTION

Molnupiravir is authorized for the treatment of mild to moderate coronavirus disease 2019 (COVID-19) in adults at high risk of progression to severe disease (1, 2). Molnupiravir is an orally administered small molecule prodrug of the ribonucleoside β-D-N4-hydroxycytidine (NHC), which has demonstrated broad preclinical activity against RNA viruses, including severe acute respiratory syndrome coronavirus 2 (SARS-CoV-2) and its variants of concern (VOC) (3 – 6). Following oral administration, the prodrug is rapidly converted to NHC, which is phosphorylated to NHC triphosphate. NHC triphosphate is a competitive alternative substrate for the virally encoded RNA-dependent RNA polymerase, and its incorporation into viral RNA misdirects the viral polymerase to incorporate either guanosine or adenosine during RNA replication. This misincorporation causes an accumulation of deleterious errors throughout the viral genome, reducing viral infectivity and replication (4, 7 – 11).

Genetic variants of SARS-CoV-2 have emerged and circulated globally throughout the COVID-19 pandemic (12). New variants are characterized predominantly by mutations in the spike protein that can impact the effectiveness of anti-SARS-CoV-2 therapeutic monoclonal antibodies and vaccines (13, 14). Due to its mechanism of action, molnupiravir is expected to remain effective against emergent viruses with novel spike protein mutations (5, 15). In previous reports, NHC was shown to be effective not only against the original wild-type SARS-CoV-2 (USA-WA1/2020) but also against early VOCs, including alpha (B.1.1.7), beta (B.1.351), and gamma (P1) in *in vitro* infection assays (16). As new SARS-CoV-2 variants emerged, we continued to evaluate the antiviral activity of NHC against more recent SARS-CoV-2 VOCs, including omicron lineages (BA.4.6, BQ.1.1, XBB.1, and XBB.1.5) (17 – 19).

NHC has also demonstrated a high barrier to the development of resistance in other RNA viruses. In previous studies with mouse hepatitis virus (coronavirus model) (20), Middle Eastern respiratory syndrome coronavirus (20), Venezuelan equine encephalitis virus (21), respiratory syncytial virus, and influenza virus (6, 22), NHC demonstrated only modest shifts (i.e., twofold) in susceptibility after prolonged exposure to the viruses *in vitro* (6, 20 – 22). It is currently unknown whether this high barrier to antiviral resistance against NHC also extends to SARS-CoV-2.

In this study, we assessed NHC antiviral activity against multiple SARS-CoV-2 variants, including omicron lineages (BA.1, BA.1.1, BA.2, BA.4, BA.4.6, BA.5, BQ.1.1, XBB.1, and XBB.1.5), alpha (B.1.1.7), beta (B.1.351), gamma (P.1), delta (B.1.617.2), lambda (C.37), and mu (B.1.621), compared with the wild-type WA1, in an *in vitro* cytopathic effect (CPE) assay. In addition, we conducted *in vitro* resistance selection studies to evaluate the potential for SARS-CoV-2 to develop resistance to NHC or a 3C-like (3CL) protease inhibitor, the results of which were anticipated to predict the emergence of drug resistance in the clinic. As part of these studies, the phenotypic and genotypic changes caused by continuous passaging of SARS-CoV-2 in the presence of NHC were evaluated, and amino acid substitutions potentially associated with viral resistance were identified.

## RESULTS

### *In vitro* antiviral activity of NHC against SARS-CoV-2 variants

In cell culture, NHC displayed potent antiviral activity against all evaluated SARS-CoV-2 omicron lineages compared with earlier variants alpha, beta, gamma, delta, lambda, and mu. The IC_50_ values ranged from 0.28 to 5.50 µM (Table 1) and were comparable (within twofold) to that against the original WA1 isolate [IC_50_ = 1.23 µM (range: 0.57–2.26)].

**TABLE 1:** Antiviral activity of SARS-CoV-2 viral isolates^*a*^

Variant	Lineage	Description	Source/BEI catalog number	Assay method (cell line)	NHC IC_50_ *^b^* (µM)	IC_50_ mean fold increase compared with WA1
WA1	A/S	SARS-CoV-2 isolate USA-WA1/2020	NR-52281	Vero E6	1.23 ± 0.60 (*n* = 17)	
Alpha	B.1.1.7	SARS-CoV-2 isolate hCoV-19/USA/CA_CDC_5574/2020	NR-54011	Vero E6	1.59 (*n* = 1)	1.29
Beta	B.1.351	SARS-CoV-2 hCoV-19/South Africa/KRISP-EC-K005321/2020	NR-54008	Vero E6	1.77 (*n* = 1)	1.44
Gamma	P.1	SARS-CoV-2 hCoV-19/Japan/TY7-501/2021	NR-54981	Vero E6	1.32 (*n* = 1)	1.07
Delta	B.1.617.2	SARS-CoV-2 isolate hCoV-19/USA/PHC658/2021	NR-55611	Vero E6	1.10, 1.68 (*n* = 2)	1.13
Omicron	BA.1	hCoV-19/USA/GA-EHC-2811C/2021	Emory University	Vero E6	1.06, 1.12 (*n* = 2)	0.89
Omicron	BA.4	hCoV-19/USA/MDHP30386/2022	NR-56806	Vero E6	2.31 ± 2.40 (*n* = 6)	1.88
Omicron	BA.2	hCoV-19/USA/CO-CDPHE-2102544747/2021	NR-56520	Vero E6	2.50, 3.40 (*n* = 2)	2.40
Omicron	BA.1.1	hCoV-19/USA/HI-CDC-4359259-001/2021	NR-56475	Vero E6	1.86, 3.35 (*n* = 2)	2.12
Omicron	BA.4.6	hCoV-19/USA/NY-CUIMCNP-11918/2022	Columbia University	Vero E6	0.57 ± 0.16 (*n* = 3)	0.46
Omicron	BA.5	SARS-CoV-2, hCoV-19/USA/COR-22-063113/2022	NR-58620	Vero-TMPRSS2-ACE2	0.55 ± 0.14 (*n* = 4)	0.45
Omicron	BQ.1.1	hCoV-19/USA/NY-CUIMCNP-12591/2022	Columbia University	Vero E6	0.76 ± 0.10 (*n* = 3)	0.62
Omicron	XBB.1	OQ362362.1	Columbia University	Vero-TMPRSS2-ACE2	0.58 ± 0.07 (*n* = 3)	0.47
Omicron	XBB.1.5	hCoV-19/USA/NY-CUIMC-NP-13300/2022	Columbia University	Vero-TMPRSS2-ACE2	0.45, 0.67 (*n* = 2)	0.46
Lambda	C.37	SARS-CoV-2 isolate hCoV-19/Peru/un-CDC-2-4069945/2021 (lineage C.37; lambda variant)	NR-55654	Vero E6	0.92, 0.98 (*n* = 2)	0.77
Mu	B.1.621	SARS-CoV-2 isolate hCoV-19/USA/WI-UW-4340/2021	University of Wisconsin	Vero E6	1.05, 1.94 (*n* = 2)	1.22

^*a*^ IC_50_, half-maximal inhibitory concentration; n, number of replicates; NHC, N-hydroxycytidine; SARS-CoV-2, severe acute respiratory syndrome coronavirus 2.

^*b*^ IC_50_ values represented as mean ± standard deviation for *n* > 2 replicates.

### *In vitro* susceptibility of SARS-CoV-2 to NHC following 30 passages

**Fig 1:**
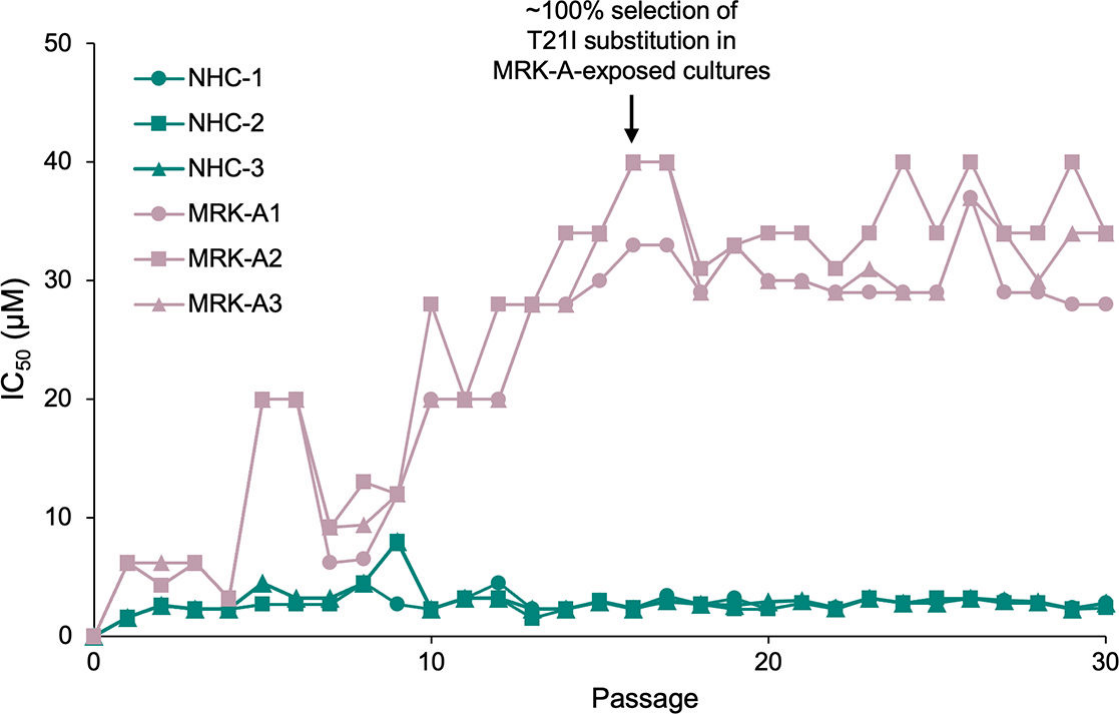
IC_50_ of SARS-CoV-2 at each passage in cultures exposed to NHC or MRK-A. MRK-A/NHC-1, 2, and 3 denote the triplicate cultures. IC_50_, half-maximal inhibitory concentration; MRK-A, 3C-like protease inhibitor; NHC, N-hydroxycytidine.

The NHC IC_50_ values against serial-passaged SARS-CoV-2 WA1 remained generally unchanged (less than 2.5-fold change) at each of the 30 passages, for all triplicate cultures (Fig. 1). In contrast, the susceptibility of SARS-CoV-2 WA1 to the control protease inhibitor, MRK-A, declined incrementally after several passages with an approximately 30-fold increase in IC_50_ values by passage 16 and beyond (Fig. 1).

To confirm the observed results from the initial IC_50_ estimates performed during the passaging experiment, susceptibility assessments were repeated for virus cultures from passages 5, 9, 15, 20, 25, and 30 using the Vero E6 cell (a subclone of Vero cells expressing high levels of ACE2; ATCC, Manassas, VA, USA) inhibitory assay with CPE scoring and with quantitative reverse transcription polymerase chain reaction (RT-PCR) method (data not shown). The results of this re-analysis confirmed that there were no increases in the NHC IC_50_ values against any of the passaged viruses evaluated over the 30 passages (data not shown).

### Genotypic characterization of passaged virus: analysis of viral sequence changes detected across the SARS-CoV-2 genome

Next-generation sequencing was performed on viral RNA from each replicate culture at each passage, and sequence changes relative to the original WA1 isolate were determined. As expected, viruses cultured in the presence of NHC accumulated nucleotide substitution variants throughout the viral genome (Fig. S1), which in turn caused random amino acid changes following 30 passages in culture (Table 2). Most nucleotide changes observed in NHC cultures were transitions, not transversions, consistent with the mechanism of action of NHC (Table 3). Although some amino acid substitutions were observed in the viral polymerase protein (nsp12) (Table 2), all substitutions were unique, and none were observed in more than one culture. Changes in the viral spike protein were noted in multiple replicates, including W64R, R682Q/W, K814R, and K986E. Currently, none of these substitutions are known to be associated with changes in antibody binding or viral infectivity. In contrast, viruses passaged in the presence of the MRK-A protease inhibitor were all rapidly selected for a T21I mutation in the viral nsp5 (3CL) protease after 10 passages and were fixed in the population by passage 30 (Table 2).

**TABLE 2:** SARS-CoV-2 amino acid changes detected at >90% frequency at passage 30 in cultures exposed to NHC or MRK-A^*a*^ ^,*d*,*e*^

Gene region	Control		NHC			MRK-A		
Replicate	1	2	1	2	3	1	2	3
Leader protein	–	–	–	S142L	E2G	–	–	–
nsp2	–	–	–	I293V, S358L, V480I	R52C, P125L, V157I, C326Y, A375T, E452K	–	–	–
nsp3	–	–	K610E, Q842R, Y1185C, C1223R, T1446A, G1681D	E115G, G334N, V453I, A655V, M953I, V1385I, I1412M, G1433S, R1518G, K1861R	G47D, V170I, V348I, I388T, S400G, K497R, N1146S, D1208N, S1406F, I1413T, V1770I, S1782L, F1823S	L1870F	–	E1799A
nsp4	–	L438R	S163F	I275V, A446V, S483F	F24L, E42G, T60I	–	–	–
3C-like proteinase	–	–	–	T24I,^*b*^ Q69R, I259V	–	**T21I** ^*b*^	**T21I,** ^*b*^ T304I	**T21I,** ^*b*^ L27V
nsp6	–	–	V289I	F55S, K263R	T181I	–	–	L260F
nsp7	–	–	–	A65T	–	–	–	–
nsp8^*c*^	–	–	K37R	–	K196R	–	–	T141M
nsp9	–	–	–	–	D26N	–	–	–
nsp10	Q36R	–	–	A104T, L138F	–	–	–	–
RNA-dependent RNA polymerase^*c*^	–	–	Y80C, H613Y	T85I, V166I, R457C, D717G	A199V, T319I, S451G, V675I	E744K	–	–
Helicase^*c*^	–	–	H290Y	V154I, V266I, V397A	D56G, T599I	–	L280F	G439E
3′-to-5′ exonuclease	–	–	A353V, V460M, S503L	G44S	K47R, I201M, P203S, V317I	–	–	–
endoRNAse	–	–	I280M	G17R	V51A, T285A	–	–	–
2′-O-Ribose methyltransferase	–	–	–	–	V96I, I171V	–	–	–
Surface glycoprotein	H245R,S247R	IHVSGTNGT68- 76X, R683W	IHVSGTNGT68- 76X, V83I, R682Q, S686N, **K986E**, V1060I, D1259N	T20I, **W64R**, N74K, M177I, T572I, **K814R**, **K986E**, G1171S	**W64R**, H66R, V127I, H207Y, T250A, R682W, **K814R**, N969S, **K986E**, F1121V, T1136I	N74K	IHVSGTNGT68-76X	L179S, E180K, S247R, S982L
ORF3a protein	–	–	F120S	T34M, I62T, I179T, Q213R, E226G, H243Y	V48I, V55I, I123M, S216L	–	–	–
Envelope protein	–	–	–	S67F	V24M	–	–	–
Membrane glycoprotein	–	H125Y	–	–	K162R	–	–	H125Y
ORF6 protein	–	–	–	Q8R	I14V, D30N	–	–	*62YSWH*
ORF7a protein	–	–	A105T	–	I107M	–	–	*
ORF7b	–	–	–	–	A15T	–	L14Sfs*30	–
ORF8 protein	–	–	W45*, E110K	–	Y42H, L57S	–	–	–
Nucleocapsid phosphoprotein	G215V	T205I	Q272R, P383S	S33G	G34E, S187L, M322I, T334I, V350I, D371N, Q380R	–	–	–
ORF10 protein	–	–	–	–	L37F	–	–	–

^*a*^ MRK-A, 3C-like protease inhibitor; NHC, N-hydroxycytidine; nsp, nonstructural protein.

^*b*^ The T24I/T21I substitution in nsp5 is involved in substrate binding to the 3C-like protease.

^*c*^ Proteins involved in viral replication.

^*d*^ Bolding indicates a change observed in more than one culture. MRK-A/NHC-1, 2, and 3 denote the triplicate cultures.

^*e*^ ”-” indicates no amino acid changes detected.

**TABLE 3:** Numbers of transitions and transversions in NHC- and MRK-A-passaged cultures^*a*^ *^,b,c^*

	Transitions				Transversions							
Replicate	C→U	G→A	U→C	A→G	G→U	U→A	A→C	C→G	U→G	A→U	C→A	G→C
Control												
1	12	0	3	3	2	1	2	1	2	0	0	0
2	8	0	0	0	1	0	1	0	3	0	0	0
NHC												
1	53	57	103	90	1	0	0	0	0	0	0	0
2	59	62	63	78	0	2	0	0	0	0	0	0
3	51	41	53	72	0	0	0	1	1	0	0	0
MRK-A												
1	13	1	6	4	2	2	2	0	1	0	1	0
2	6	0	2	8	1	0	1	0	0	0	1	0
3	6	2	1	0	0	1	2	1	0	0	0	0

^*a*^ A, adenine; C, cytosine; G, guanine; MRK-A, 3C-like protease inhibitor; NHC, N-hydroxycytidine; U, uracil.

^*b*^ MRK-A/NHC-1, 2, and 3 denote the triplicate cultures.

^*c*^ Nucleotide changes detected at ≥10% frequency at passage 30.

### Phenotypic characterization of passaged viruses: viral growth kinetics and susceptibility to neutralizing antibodies

Viral isolates from passages 5, 9, 15, 20, 25, and 30 of untreated control and NHC cultures were assessed for viral growth kinetics on Vero E6 cells. The relative slope of the viral growth curve during exponential growth is plotted for NHC-treated and control (no-drug) cultures (Fig. S2). Following five passages, both control virus and NHC-passaged cultures showed an increase in viral replication compared with the original unpassaged WA1 isolate in Vero E6 cells. This increased replication is likely due to tissue culture adaptation of the virus because it occurred in both the control and the NHC-treated cultures. However, additional passaging did not result in further enhancement of viral replication capacity in either NHC or control cultures (Fig. S2).

**Fig 2:**
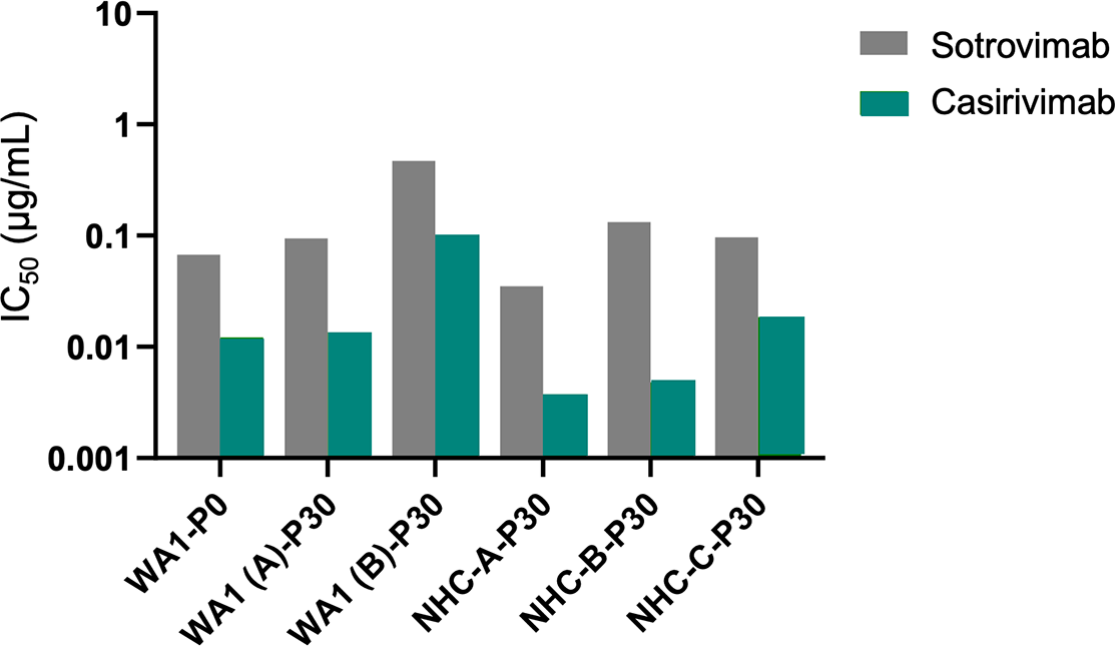
*In vitro* susceptibility of two neutralizing antibodies to NHC-passaged and no drug control–passaged WA1 viruses. NHC-A, B, and C denote triplicate cultures. WA1 (A) and (B) denote duplicate cultures. IC_50_, half-maximal inhibitory concentration; NHC, N-hydroxycytidine; P, passage number; WA1, SARS-CoV-2 isolate USA-WA1/2020.

To determine if amino acid substitutions observed in the spike protein following passaging with NHC affected neutralizing antibodies, viral isolates from NHC-treated and control SARS-CoV-2 WA1 passage 30 cultures were tested for susceptibility to two neutralizing antibodies, S309 (sotrovimab) and REGN10933 (casirivimab), targeting the SARS-CoV-2 spike protein. Results showed that NHC-passaged viruses remained as susceptible to both neutralizing antibodies as the passaged control virus (Fig. 2).

## DISCUSSION

NHC was effective against all evaluated SARS-CoV-2 omicron lineages (BA.1, BA.1.1, BA.2, BA.4, BA.4.6, BA.5, BQ.1.1, XBB.1, and XBB.1.5), B1.1.7 (alpha), B1.351 (beta), P.1 (gamma), C.37 (lambda), and B.1.621 (mu). These results are consistent with published studies evaluating NHC against SARS-CoV-2 omicron (B.1.1.529 and BA.2) and other variants (alpha [B.1.1.7], beta [B.1.351], gamma [P.1], delta [B.1.617.2], and B1.13) (5, 23 – 26). The susceptibility of the passaged SARS-CoV-2 to the control inhibitor, MRK-A, declined incrementally over 30 passages, confirming the utility of this method for resistance selection. In contrast, the susceptibility of SARS-CoV-2 to inhibition by NHC did not significantly change over 30 passages, as demonstrated by the NHC IC_50_ values of less than twofold change from baseline. This finding is consistent with data from studies of several other RNA viruses demonstrating a high barrier to resistance against NHC (6, 21, 22).

This high barrier to resistance suggests that molnupiravir should have long-term clinical utility against current and future variants (thus far, primarily differentiated by changes in the spike protein). This assumption is further supported by our *in vitro* data confirming NHC activity against omicron and other variants, indicating the potential of molnupiravir for the treatment of existing and future SARS-CoV-2 variants, including multiple omicron lineages.

Sequence analysis of the passaged virus confirmed the mechanism of action of molnupiravir. Each culture passaged with NHC showed an accumulation of random amino acid changes across the genome over time, which were not associated with changes in viral susceptibility to NHC. For NHC-passaged cultures, no amino acid substitutions in the viral replicase gene products were observed in more than one culture; however, several amino acid substitutions were observed in two or more cultures for the spike protein, that is, W64R, R682Q/W, K814R, and K986E. Currently, none of these substitutions are known to be associated with changes in antibody binding or viral infectivity (27, 28). Moreover, following 30 passages with NHC, viruses from all three NHC-treated cultures remained susceptible to several monoclonal antibodies. In this study, passaging experiments were conducted with only SARS-CoV-2 wild-type virus but not with more recent variants. Therefore, the effect of existing mutations in currently circulating isolates on the resistance development could not be determined. However, all SARS-CoV-2 variants we examined to date demonstrated an equivalent sensitivity to molnupiravir in cell culture. Furthermore, existing mutations in RNA-dependent RNA polymerase (nsp12) in the variant viruses do not overlap with those developed over passage.

To assess the impact of accumulated amino acid substitutions on viral growth, replication capacity studies were performed. The results of these studies demonstrated that viral replication kinetics increased after five passages in both the control and NHC cultures. This result is likely due to tissue culture adaptation of the virus during initial rounds of passaging and not due to the impact of NHC as no further increase in replication kinetics was observed during subsequent passaging despite the accumulation of additional amino acid substitutions in the NHC cultures. To determine if amino acid substitutions observed in the spike protein following passaging with NHC affected neutralizing antibodies, viral isolates from NHC and SARS-CoV-2 WA1 passage 30 cultures were tested for susceptibility to two neutralizing antibodies, sotrovimab and casirivimab, targeting the SARS-CoV-2 spike protein. Sotrovimab neutralizes SARS-CoV-2 by binding to a strongly conserved epitope outside the receptor-binding motif (29). Casirivimab neutralizes SARS-CoV-2 by binding to the “up” and “up and down” conformation of the viral receptor–binding domain (30). In this study, NHC-passaged viruses remained as susceptible as the control virus (SARS-CoV-2 WA1) to both neutralizing antibodies, which further suggests that the amino acid substitutions observed did not impact the activity of neutralizing antibodies. A T21I amino acid substitution in nsp5 was noted in all MRK-A triplicate cultures. This substitution is involved in substrate binding to the 3CL protease, which is the main protease involved in the replication cycle in SARS-CoV-2 (31). Of note, this same substitution was recently shown to contribute to resistance to nirmatrelvir during *in vitro* resistance studies and likely contributed to the loss of susceptibility to the MRK-A compound in these cultures (32). However, additional phenotyping studies are required to confirm the impact of this mutation on the susceptibility of SARS-CoV-2 to MRK-A.

In conclusion, the SARS-CoV-2 data presented here are consistent with previous studies with other RNA viruses and further support the conclusion that molnupiravir/NHC has a high barrier to the development of SARS-CoV-2 resistance, which is expected to translate to durable clinical efficacy. In addition, NHC was effective against all evaluated SARS-CoV-2 variants *in vitro*, further demonstrating that changes in the viral spike proteins associated with novel variants do not impact antiviral activity of NHC.

## MATERIALS AND METHODS

### Evaluation of NHC antiviral activity against SARS-CoV-2 variants

The antiviral activity of NHC against the ancestral WA1 isolate and multiple SARS-CoV-2 variants (omicron lineages [B.1.1.529, BA.1.1, BA.2, BA.4.6, BQ.1.1, XBB.1, and XBB.1.5], alpha [B.1.1.7], beta [B.1.351], gamma [P.1], delta [B.1.617.2], lambda [C.37], and mu [B.1.621]) was evaluated by assessing the ability of NHC to reduce viral CPEs in Vero E6 cells. All viruses were propagated in Vero E6 cells for one passage. Virus infectious titer for each stock was determined by an end point dilution CPE assay on Vero E6 cells as previously described (33). Antiviral assays were initially performed at Columbia University Medical Center using previously described methods (32, 34). More recently, assays were conducted at the Research Laboratories of Merck & Co., Inc., Rahway, NJ, USA, where protection from virus-induced CPE was measured using the CellTiter-Glo 2.0 cell viability assay kit (Promega, Madison, WI, USA) to assess antiviral activity of NHC against SARS-CoV-2 variants WA1 and omicron lineages BA.4 and BA.5 in Vero E6/TMPRSS2 cells (BPS Bioscience, San Diego, CA, USA). Prior to assay start, Vero E6/TMPRSS2 cells were removed from maintenance flasks, counted, and diluted in assay media to 4.0 × 10^4^ cells/mL. Prepared cells were placed into conical tubes for bulk infection with each SARS-CoV-2 isolate at a targeted multiplicity of infection (MOI) of 0.1. serial dilutions (12 points) of compounds (NHC or remdesivir) were dispensed into 384-well assay plates with the highest final drug concentration of 10 µM. Dimethyl sulfoxide (DMSO)-only (no drug control) wells were also prepared. After bulk infection, 50 µL (2,000 cells) of the infected cells was added to each well of the assay plates. Uninfected cells (50 µL at 4.0 × 10^4^ cells/mL, i.e., 2,000 cells per well) were added to designated wells on the assay plates; these served as the minimum CPE control, whereas DMSO-treated infected cells served as the maximum CPE control. The assay plates were then incubated in a humidified chamber at 37°C, 5% carbon dioxide, for 3–4 days.

Cell viability in compound-treated wells was assessed using the CellTiter-Glo 2.0 kit (Promega, Madison, WI, USA) as per the manufacturer’s protocol. The assay signal (luminescence) was read using an EnSight multimode plate reader (PerkinElmer, Inc., MA, USA). IC_50_ values were calculated with a four-parameter, variable-slope dose–response curve fit of the assay data for each compound using GraphPad Prism 8.0 software (GraphPad Software Inc., San Diego, CA, USA).

### *In vitro* selection and characterization of SARS-CoV-2 resistance to NHC versus a protease inhibitor

Prior to conducting, this study was reviewed and approved by the Columbia University Institutional Biosafety Committee (IBC) under appendices APA-AQNR6252 and APA-BDYW7534. All passaging experiments were conducted by trained personnel in Biosafety Level-3 (BSL-3) laboratories at Columbia University.

An *in vitro* culture system was used to select potential SARS-CoV-2 strains with reduced susceptibility to NHC or to an experimental 3CL protease inhibitor (MRK-A). In this system, parallel virus (SARS-CoV-2, US WA1/2020) cultures were serially passaged in cell culture in the presence of varying concentrations (0–100 µM) of NHC or MRK-A, and the development of drug resistance was monitored for each passage of culture. The passaged virus cultures were characterized phenotypically (i.e., drug susceptibility, viral growth kinetics, and susceptibility to neutralizing antibodies) and genotypically (i.e., sequencing of viral RNA to identify changes in viral proteins potentially associated with resistance).

### Resistance selection studies

The SARS-CoV-2 antiviral resistance selection assay was performed as a variation of the assay first described by Iketani et al (32, 34). The assay was performed by monitoring SARS-CoV-2 CPE on infected Vero E6 cells to determine the compound IC_50_ values and changes in drug susceptibility. Vero E6 cells were plated in 24-well plates at 1 × 10^5^ cells/mL per well on the day prior to inoculation with the virus. Independent triplicate cultures (series A, B, and C) were infected with SARS-CoV-2 (WA1 strain) in the presence of different concentrations of NHC, MRK-A, or culture medium alone at the final volume of 1.5 mL/well. SARS-CoV-2 was inoculated into each well at 50% tissue culture infectious dose (TCID_50_) of 5,000 units for the initial culture or using 100 µL of the supernatant from the previous passage culture. NHC or MRK-A (dissolved in 100 mM DMSO) was added to the cell culture across a range of concentrations in a threefold dilution series based on the IC_50_ of each drug, that is, 27 × IC_50_, 9 × IC_50_, 3 × IC_50_, 1 × IC_50_, 0.33 × IC_50_, and 0.1 × IC_50_ (see Fig. S3 for a schema of the experimental setup). SARS-CoV-2 cultures passaged in the absence of inhibitor were maintained in two wells to serve as a passage control and define genetic changes and tissue culture adaptation without the drug selective pressure. After the addition of the virus, cells were incubated at 37°C and 5% CO_2_ for 72 hours.

Viral replication as measured by CPE was observed daily using a Revolve microscope (10× objective; ECHO, San Diego, CA, USA). The scoring of raw data per well was based on the distribution of CPE per well with the following scoring scale:

(−) = no CPE

(d+) = some dead cells (no replication)

(+) = <10% CPE in well

(++) = approximately 10% to 30% CPE in well

(+++) = approximately 40% to 50% CPE in well

(++++) = approximately 60% to 100% CPE in well

After scoring, the culture supernatant (100 µL) from wells with the highest drug concentration exhibiting a CPE score of 2+ or higher (>10% CPE in well) was used to seed the next passage. The remaining supernatant was used for SARS-CoV-2 viral RNA isolation or stored at −80°C. The supernatant from wells with lower drug or control concentration (CPE score of less than 3) was stored as a back-up virus sample. Virus passaging for all samples continued up to passage 30 (NHC-p30) to select for resistance against NHC or MRK-A. When no CPE was observed, that is, when the infection was not successful, the backup virus sample from the previous passage was used to restart the culture.

### Determining changes in IC_50_ value

The IC_50_ values for NHC and MRK-A at each passage were estimated based on CPE scoring (as described previously) by the crossing point with 50% inhibition on a curve fitted using DeltaGraph (Red Rock Software, Inc., Salt Lake City, UT, USA) or by analysis using GraphPad Prism v7.0.

### Genotypic characterization of passaged virus

Viral RNA from culture supernatants was collected for each replicate culture, at each passage, for both NHC and MRK-A, and purified using the PureLink Pro 96 Viral RNA/DNA Purification Kit (ThermoFisher Scientific, Waltham, MA, USA). Whole genome SARS-CoV-2 sequencing libraries were prepared via the QIAseq DIRECT SARS-CoV-2 Kit (Qiagen, Hilden, Germany).

Sequencing was performed at the DNA Sequencing & Genotyping Center, University of Delaware, Ammon-Pinizzotto Biopharmaceutical Innovation Center (Newark, DE, USA), on the Illumina platform (San Diego, CA, USA), yielding 2 × 151 nucleotide paired-end reads. Genomic variants were identified using the following procedure: PCR primers were removed from the reads with the open-source program removePrimer (accessed via github.com/jsh58/AmpliconTools) with default parameters and “-ef 2 -fp −1,1.” Only read pairs in which both primers were identified, and subsequently removed, were further analyzed. Read pairs generated from likely PCR chimeras (having primers removed on the same strand, in the wrong orientation, or more than 1 kb apart) were also filtered out; samples with excessive (>5%) chimeric fragments were removed from further analysis. For the remaining samples, the paired reads were merged together using NGmerge v0.3 (35) with default parameters and “-d.” Reads that were successfully merged were aligned as unpaired full-length fragments by bowtie2 v2.3.5.1 (36) to the SARS-CoV-2 reference genome (GenBank ID NC_045512.2) (37). Reads that failed merging by NGmerge were aligned as regular paired-end reads by bowtie2 with default parameters and “-X 1000.” The alignment files were combined and converted to binary form by SAMtools v1.9 (38). Pileup files were produced from the alignment files, counting only bases with quality scores of at least 30. Variants from the reference genome were identified at sites with a minimum read coverage of 50 and a minimum allele fraction of 0.10. Spurious variants due to large deletions were investigated and manually corrected. Variants for multiple samples were combined into a single table and annotated for the predicted effects on SARS-CoV-2 proteins.

### Phenotypic characterization of passaged virus: viral growth kinetics and susceptibility to neutralizing antibodies

The passaged viruses were propagated in Vero E6 cells in 75 cm^2^ flasks in the absence of drugs. The supernatant of each culture with a CPE score of 3+ or higher (>40% CPE in well) was harvested through centrifugation at 2,500 rpm for 10 min (Thermo Sorvall Legend XTR Refrigerated Centrifuge; Marshall Scientific, Hampton, NH, USA), aliquoted, and stored at −80°C. The propagated viruses (cultures with a CPE score 3+ or higher) were used to characterize viral growth kinetics or fitness in Vero E6 cells (39) at specific time points (passages 0, 5, 10, 15, 20, 25, and 30), and to assess susceptibility to NHC, MRK-A, and other agents, such as neutralizing antibodies, at passage 30. The infectious titer was determined by the end point dilution culture method as described by Cresta et al. (40).

For the assessment of the replication capacity of the passaged viruses, Vero E6 cell cultures, which were plated at 1.5 × 10^4^ cells/mL per well a day before infection, were inoculated, in triplicate, with viruses from NHC-passaged culture at 200 TCID_50_ units per well. Free virions were removed by a complete change of medium at 6 hours post infection. The supernatant was collected at 11, 23.5, 32.5, and 48 hours following inoculation, and viral RNA was isolated using the PureLink Pro 96 Viral RNA/DNA Purification Kit (ThermoFisher Scientific). The viral RNA copy number was determined by reverse transcription quantitative PCR (RT-qPCR) using the TaqPath 1-Step RT-qPCR Master Mix, CG (ThermoFisher Scientific), with the CDC N1 primer and probe from the 2019-nCoV RUO Kit (Integrated DNA Technologies, Coralville, IA, USA) and using Applied Biosystems 7500 Fast Dx Real-Time PCR instrument (ThermoFisher Scientific). The ascending slope during the initial exponential growth phase on Vero E6 cells was estimated based on the increase in the viral RNA copy number in the culture supernatant.

To evaluate the susceptibility of the NHC-passaged viruses to neutralizing antibodies S309 (sotrovimab) and REGN10933 (casirivimab), Vero E6 cells were cultured in 96-well plates at 1.5 × 10^4^ cells per well overnight. The following day, virus samples were mixed with neutralizing antibody in a total volume of 100 µL (in triplicate): 1,500 TCID_50_ units of each of the three lineages (A, B, and C) of NHC-p30 (virus passaged for 30 times in the presence of NHC) along with the original virus, WA1-p0 (zero passages), and two no-drug control-passaged viruses, WA1-p30.1 and WA1-p30.2. The neutralizing antibody consisted of a fivefold dilution series starting at 10 µg/mL (i.e., 10, 2, 0.4, 0.08, 0.016, 0.0032, 0.00064, and 0 µg/mL). The virus–antibody mixture was incubated at 37°C for 1 hour and inoculated onto individual Vero E6 cell culture wells. Three days following infection, the CPE of each culture well was scored (as described previously), and the percentage of the CPE score was used to estimate the neutralizing antibody IC_50_ values using GraphPad Prism v9.4.1.

## Data Availability

All raw sequencing data are available in the National Center for Biotechnology Information (NCBI) Short Read Archive (SRA) under BioProject PRJNA1035781 (accession numbers SRR26669148 to SRR26669387).

## SUPPLEMENTAL MATERIAL

Figs S1-S3 aac.00953-23-s0001.pdf Supplemental Figures.

ASM does not own the copyrights to Supplemental Material that may be linked to, or accessed through, an article. The authors have granted ASM a non-exclusive, world-wide license to publish the Supplemental Material files. Please contact the corresponding author directly for reuse.

## References

[1] Anonymous . 2021. Merck Sharp & Dohme (UK) Limited, London, UK. Lagevrio 200 Mg Hard Capsules: UK Prescribing Information. Available from: https://www.gov.uk/government/publications/regulatory-approval-of-lagevrio-molnupiravir/summary-of-product-characteristics-for-lagevrio

[2] U.S. Food and Drug Administration . 2022. Fact sheet for Healthcare providers: emergency use authorization for lagevrio (molnupiravir) capsules

[3] Saito A , Tamura T , Zahradnik J , Deguchi S , Tabata K , Anraku Y , Kimura I , Ito J , Yamasoba D , Nasser H , et al. . 2022. Virological characteristics of the SARS-CoV-2 Omicron BA.2.75 variant. Cell Host Microbe 30:1540–1555. doi:10.1016/j.chom.2022.10.003 36272413 PMC9578327

[4] Sheahan TP , Sims AC , Zhou S , Graham RL , Pruijssers AJ , Agostini ML , Leist SR , Schäfer A , Dinnon KH , Stevens LJ , et al. . 2020. An orally bioavailable broad-spectrum antiviral inhibits SARS-CoV-2 in human airway epithelial cell cultures and multiple coronaviruses in mice. Sci Transl Med 12:eabb5883. doi:10.1126/scitranslmed.abb5883 32253226 PMC7164393

[5] Takashita E , Yamayoshi S , Fukushi S , Suzuki T , Maeda K , Sakai-Tagawa Y , Ito M , Uraki R , Halfmann P , Watanabe S , Takeda M , Hasegawa H , Imai M , Kawaoka Y . 2022. Efficacy of antiviral agents against the Omicron Subvariant BA.2.75. N Engl J Med 387:1236–1238. doi:10.1056/NEJMc2209952 36121928 PMC9511631

[6] Yoon JJ , Toots M , Lee S , Lee ME , Ludeke B , Luczo JM , Ganti K , Cox RM , Sticher ZM , Edpuganti V , Mitchell DG , Lockwood MA , Kolykhalov AA , Greninger AL , Moore ML , Painter GR , Lowen AC , Tompkins SM , Fearns R , Natchus MG , Plemper RK . 2018. Orally efficacious broad-spectrum ribonucleoside analog inhibitor of influenza and respiratory syncytial viruses. Antimicrob Agents Chemother 62:e00766-18. doi:10.1128/AAC.00766-18 29891600 PMC6105843

[7] Kabinger F , Stiller C , Schmitzová J , Dienemann C , Kokic G , Hillen HS , Höbartner C , Cramer P . 2021. mechanism of molnupiravir-induced SARS-CoV-2 mutagenesis. Nat Struct Mol Biol 28:740–746. doi:10.1038/s41594-021-00651-0 34381216 PMC8437801

[8] Tian L , Pang Z , Li M , Lou F , An X , Zhu S , Song L , Tong Y , Fan H , Fan J . 2022. Molnupiravir and its antiviral activity against COVID-19. Front Immunol 13:855496. doi:10.3389/fimmu.2022.855496 35444647 PMC9013824

[9] Gordon CJ , Tchesnokov EP , Schinazi RF , Götte M . 2021. Molnupiravir promotes SARS-CoV-2 Mutagenesis via the RNA template. J Biol Chem 297:100770. doi:10.1016/j.jbc.2021.100770 33989635 PMC8110631

[10] Jayk Bernal A , Gomes da Silva MM , Musungaie DB , Kovalchuk E , Gonzalez A , Delos Reyes V , Martín-Quirós A , Caraco Y , Williams-Diaz A , Brown ML , Du J , Pedley A , Assaid C , Strizki J , Grobler JA , Shamsuddin HH , Tipping R , Wan H , Paschke A , Butterton JR , Johnson MG , De Anda C . 2022. Molnupiravir for oral treatment of COVID-19 in nonhospitalized patients. N Engl J Med 386:509–520. doi:10.1056/NEJMoa2116044 34914868 PMC8693688

[11] Painter WP , Holman W , Bush JA , Almazedi F , Malik H , Eraut NCJE , Morin MJ , Szewczyk LJ , Painter GR . 2021. Human safety, tolerability, and pharmacokinetics of molnupiravir, a novel broad-spectrum oral antiviral agent with activity against SARS-CoV-2. Antimicrob Agents Chemother 65:e02428-20. doi:10.1128/AAC.02428-20 33649113 PMC8092915

[12] World Health Organization . 2022. Tracking SARS-CoV-2 Variants. Available from: https://www.who.int/activities/tracking-SARS-CoV-2-variants

[13] VanBlargan LA , Errico JM , Halfmann PJ , Zost SJ , Crowe JE , Purcell LA , Kawaoka Y , Corti D , Fremont DH , Diamond MS . 2022. An infectious SARS-CoV-2 B.1.1.529 Omicron virus escapes neutralization by therapeutic monoclonal antibodies. Nat Med 28:490–495. doi:10.1038/s41591-021-01678-y 35046573 PMC8767531

[14] Yaqinuddin A , Shafqat A , Kashir J , Alkattan K . 2021. Effect of SARS-CoV-2 mutations on the efficacy of antibody therapy and response to vaccines. Vaccines (Basel) 9:914. doi:10.3390/vaccines9080914 34452039 PMC8402590

[15] Takashita E , Kinoshita N , Yamayoshi S , Sakai-Tagawa Y , Fujisaki S , Ito M , Iwatsuki-Horimoto K , Chiba S , Halfmann P , Nagai H , Saito M , Adachi E , Sullivan D , Pekosz A , Watanabe S , Maeda K , Imai M , Yotsuyanagi H , Mitsuya H , Ohmagari N , Takeda M , Hasegawa H , Kawaoka Y . 2022. Efficacy of antibodies and antiviral drugs against COVID-19 Omicron variant. N Engl J Med 386:995–998. doi:10.1056/NEJMc2119407 35081300 PMC8809508

[16] Grobler J , Strizki J , Murgolo N , Gao W , Cao Y , Zhang Y , Du J , Nair M , Huang Y , Luo Y , Hazuda D , Ho DD , Ho DD . 2021. Molnupiravir maintains antiviral activity against SARS-CoV-2 variants in vitro and in early clinical studies. Open Forum Infect Dis 8:S373–S373. doi:10.1093/ofid/ofab466.742

[17] Centers for Disease Control and Prevention . 2023. COVID Data Tracker. Available from: https://covid.cdc.gov/covid-data-tracker/#variant-proportions. Retrieved Jun 2023.

[18] Wang Q , Iketani S , Li Z , Liu L , Guo Y , Huang Y , Bowen AD , Liu M , Wang M , Yu J , Valdez R , Lauring AS , Sheng Z , Wang HH , Gordon A , Liu L , Ho DD . 2023. Alarming antibody evasion properties of rising SARS-CoV-2 BQ and XBB Subvariants. Cell 186:279–286. doi:10.1016/j.cell.2022.12.018 36580913 PMC9747694

[19] Miller J , Hachmann NP , Collier AY , Lasrado N , Mazurek CR , Patio RC , Powers O , Surve N , Theiler J , Korber B , Barouch DH . 2023. Substantial neutralization escape by SARS-CoV-2 Omicron variants BQ.1.1 and XBB.1. N Engl J Med 388:662–664. doi:10.1056/NEJMc2214314 36652339 PMC9878581

[20] Agostini ML , Pruijssers AJ , Chappell JD , Gribble J , Lu X , Andres EL , Bluemling GR , Lockwood MA , Sheahan TP , Sims AC , Natchus MG , Saindane M , Kolykhalov AA , Painter GR , Baric RS , Denison MR . 2019. Small-molecule antiviral β-d-N(4)-hydroxycytidine inhibits a proofreading-intact coronavirus with a high genetic barrier to resistance. J Virol 93:e01348-19. doi:10.1128/JVI.01348-19 31578288 PMC6880162

[21] Urakova N , Kuznetsova V , Crossman DK , Sokratian A , Guthrie DB , Kolykhalov AA , Lockwood MA , Natchus MG , Crowley MR , Painter GR , Frolova EI , Frolov I . 2018. β-d-N(4)-hydroxycytidine is a potent anti-alphavirus compound that induces a high level of mutations in the viral genome. J Virol 92. doi:10.1128/JVI.01965-17 PMC5774879 29167335

[22] Toots M , Yoon JJ , Cox RM , Hart M , Sticher ZM , Makhsous N , Plesker R , Barrena AH , Reddy PG , Mitchell DG , Shean RC , Bluemling GR , Kolykhalov AA , Greninger AL , Natchus MG , Painter GR , Plemper RK . 2019. Characterization of orally efficacious influenza drug with high resistance barrier in ferrets and human airway epithelia. Sci Transl Med 11:eaax5866. doi:10.1126/scitranslmed.aax5866 31645453 PMC6848974

[23] Bojkova D , Stack R , Rothenburger T , Kandler JD , Ciesek S , Wass MN , Michaelis M , Cinatl J . 2022. Synergism of interferon-beta with antiviral drugs against SARS-CoV-2 variants. J Infect 85:573–607. doi:10.1016/j.jinf.2022.07.023 PMC9339084 35917841

[24] Dabrowska A , Szczepanski A , Botwina P , Mazur-Panasiuk N , Jiřincová H , Rabalski L , Zajic T , Popowicz G , Pyrc K . 2021. Efficacy of antiviral drugs against the Omicron variant of SARS-CoV-2. bioRxiv. doi:10.1101/2021.12.21.473268:2021.12.21.473268

[25] Rosales R , McGovern BL , Rodriguez ML , Rai DK , Cardin RD , Anderson AS , Sordillo EM , Bakel H , Simon V , García-Sastre A , White KM . 2022. Nirmatrelvir, molnupiravir, and remdesivir maintain potent. bioRxiv. doi:10.1101/2022.01.17.476685:2022.01.17.476685

[26] Vangeel L , Chiu W , De Jonghe S , Maes P , Slechten B , Raymenants J , André E , Leyssen P , Neyts J , Jochmans D . 2022. Remdesivir, molnupiravir and nirmatrelvir remain active against SARS-CoV-2 Omicron and other variants of concern. Antiviral Res 198:105252. doi:10.1016/j.antiviral.2022.105252 35085683 PMC8785409

[27] Li Q , Wu J , Nie J , Zhang L , Hao H , Liu S , Zhao C , Zhang Q , Liu H , Nie L , Qin H , Wang M , Lu Q , Li X , Sun Q , Liu J , Zhang L , Li X , Huang W , Wang Y . 2020. The impact of mutations in SARS-CoV-2 spike on viral infectivity and antigenicity. Cell 182:1284–1294. doi:10.1016/j.cell.2020.07.012 32730807 PMC7366990

[28] Zhou W , Xu C , Wang P , Anashkina AA , Jiang Q . 2022. Impact of mutations in SARS-CoV-2 spike on viral infectivity and antigenicity. Brief Bioinform 23:bbab375. doi:10.1093/bib/bbab375 34518867 PMC8499914

[29] Liu L , Iketani S , Guo Y , Chan JFW , Wang M , Liu L , Luo Y , Chu H , Huang Y , Nair MS , Yu J , Chik KKH , Yuen TTT , Yoon C , To KKW , Chen H , Yin MT , Sobieszczyk ME , Huang Y , Wang HH , Sheng Z , Yuen K-Y , Ho DD . 2022. Striking antibody evasion manifested by the Omicron variant of SARS-CoV-2. Nature 602:676–681. doi:10.1038/s41586-021-04388-0 35016198

[30] Nguyen H , Lan PD , Nissley DA , O’Brien EP , Li MS . 2022. Cocktail of REGN antibodies binds more strongly to SARS-CoV-2 than its components, but the Omicron variant reduces its neutralizing ability. J Phys Chem B 126:2812–2823. doi:10.1021/acs.jpcb.2c00708 35403431 PMC9016775

[31] Yashvardhini N , Kumar A , Jha DK . 2022. Analysis of SARS-CoV-2 mutations in the main viral protease (NSP5) and its implications on the vaccine designing strategies. Vacunas 23:S1–S13. doi:10.1016/j.vacun.2021.10.002 PMC8639442 34876891

[32] Iketani S , Mohri H , Culbertson B , Hong SJ , Duan Y , Luck MI , Annavajhala MK , Guo Y , Sheng Z , Uhlemann AC , Goff SP , Sabo Y , Yang H , Chavez A , Ho DD . 2023. Multiple pathways for SARS-CoV-2 resistance to nirmatrelvir. Nature 613:558–564. doi:10.1038/s41586-022-05514-2 36351451 PMC9849135

[33] Liu Z , Zheng H , Lin H , Li M , Yuan R , Peng J , Xiong Q , Sun J , Li B , Wu J , Yi L , Peng X , Zhang H , Zhang W , Hulswit RJG , Loman N , Rambaut A , Ke C , Bowden TA , Pybus OG , Lu J , Pfeiffer JK . 2020. Identification of common deletions in the spike protein of severe acute respiratory syndrome coronavirus 2. J Virol 94. doi:10.1128/JVI.00790-20 PMC7431800 32571797

[34] Iketani S , Forouhar F , Liu H , Hong SJ , Lin FY , Nair MS , Zask A , Huang Y , Xing L , Stockwell BR , Chavez A , Ho DD . 2021. Lead compounds for the development of SARS-CoV-2 3CL protease inhibitors. Nat Commun 12:2708. doi:10.1038/s41467-021-23082-3 33795671 PMC8016852

[35] Gaspar JM . 2018. NGmerge: merging paired-end reads via novel empirically-derived models of sequencing errors. BMC Bioinformatics 19:536. doi:10.1186/s12859-018-2579-2 30572828 PMC6302405

[36] Langmead B , Salzberg SL . 2012. Fast gapped-read alignment with Bowtie 2. Nat Methods 9:357–359. doi:10.1038/nmeth.1923 22388286 PMC3322381

[37] National Center for Biotechnology Information . 2020. Severe acute respiratory syndrome Coronavirus 2 isolate Wuhan-Hu-1, complete genome. Available from: https://www.ncbi.nlm.nih.gov/nuccore/NC_045512.2

[38] Danecek P , Bonfield JK , Liddle J , Marshall J , Ohan V , Pollard MO , Whitwham A , Keane T , McCarthy SA , Davies RM , Li H . 2021. Twelve years of SAMtools and BCFtools. Gigascience 10:giab008. doi:10.1093/gigascience/giab008 33590861 PMC7931819

[39] Ikhlas S , Usman A , Kim D , Cai D . 2022. Exosomes/microvesicles target SARS-CoV-2 via innate and RNA-induced immunity with PIWI-piRNA system. Life Sci Alliance 5:e202101240. doi:10.26508/lsa.202101240 34862272 PMC8645330

[40] Cresta D , Warren DC , Quirouette C , Smith AP , Lane LC , Smith AM , Beauchemin CAA . 2021. Time to revisit the endpoint dilution assay and to replace the TCID50 as a measure of a virus sample’s infection concentration. PLoS Comput Biol 17:e1009480. doi:10.1371/journal.pcbi.1009480 34662338 PMC8553042

