# Supplementary material for: Molnupiravir maintains antiviral activity against SARS-CoV-2 variants and exhibits a high barrier to the development of resistance
Source: Antimicrob Agents Chemother. 2023 Dec 4;68(1):e00953-23. doi: 10.1128/aac.00953-23 (PMC10777856; doi:10.1128/aac.00953-23)
Supplement: Figs S1-S3 — Supplemental Figures. [file aac.00953-23-s0001.pdf]

## Supplementary

**FIG S1** Cumulative substitution variants in SARS-CoV-2 at each passage in cultures exposed to NHC and MRK-A.[a]

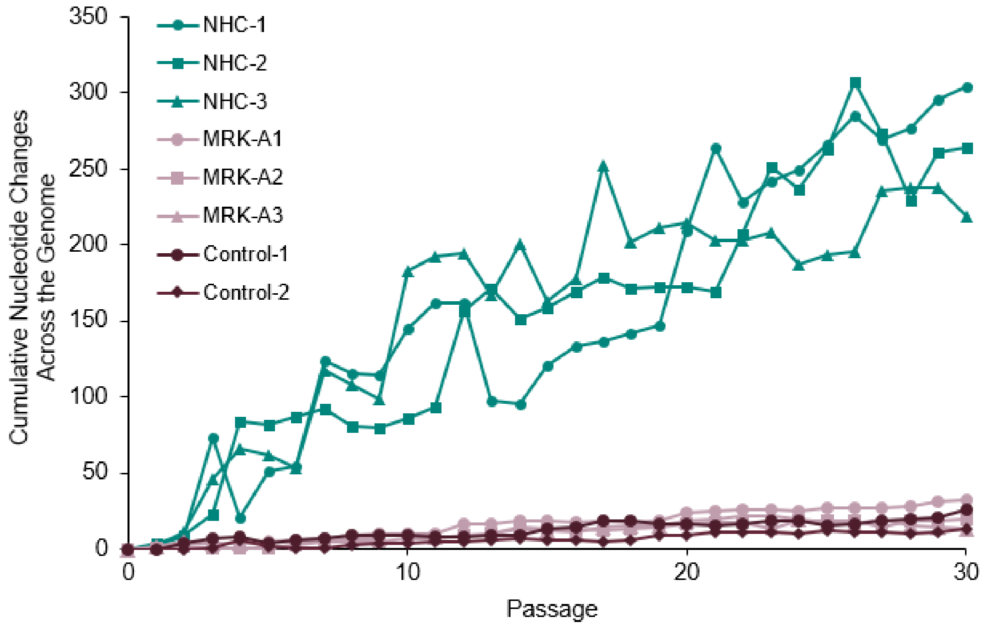


[a]Nucleotide changes detected at ≥10% frequency.

MRK-A/NHC-1, 2, and 3 denote the triplicate cultures.

MRK-A, 3C-like protease inhibitor; NHC, N-hydroxycytidine.

**FIG S2** Changes in the initial exponential growth phase of NHC-passaged viruses and no drug control–passaged WA1 viruses in Vero E6 cells.

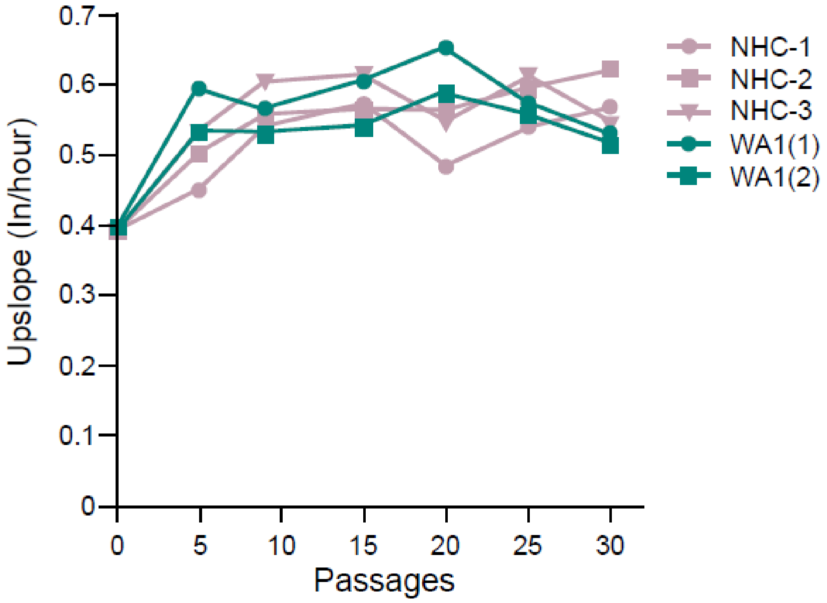


NHC-1, 2, and 3 denote triplicates of NHC-passaged cultures. WA1 (1) and (2) denote duplicates of no drug–control passaged WA1 cultures.

The passaged viruses were inoculated onto Vero E6 cell cultures, and the upslope during the initial exponential growth phase was estimated based on the viral RNA copy number in the culture supernatant.

ln, logarithm of viral replication (slope); NHC, N-hydroxycytidine; WA1, SARS-CoV-2 Isolate USA-WA1/2020.

**FIG S3** SARS-CoV-2 passaging plate configuration.

| | Test article/control concentration | | | | | |
|---|---|---|---|---|---|---|
| | 27X $IC_{50}$<br>1 | 9X $IC_{50}$<br>2 | 3X $IC_{50}$<br>3 | 1X $IC_{50}$<br>4 | 0.33X $IC_{50}$<br>5 | 0.1X $IC_{50}$<br>6 |
| A | A1 | A2 | A3 | A4 | A5 | A6 |
| B | B1 | B2 | B3 | B4 | B5 | B6 |
| C | C1 | C2 | C3 | C4 | C5 | C6 |
| D | A<br>(no drug) | B<br>(no drug) | C<br>(no drug) | WA1 | WA1 | no<br>virus |

A, B, and C denote the triplicate cultures.

$IC_{50}$, half-maximal inhibitory concentration; WA1, SARS-CoV-2 WA1/2020 strain virus (control).
